# Generative deep learning furthers the understanding of local distributions of fat and muscle on body shape and health using 3D surface scans

**DOI:** 10.1038/s43856-024-00434-w

**Published:** 2024-01-30

**Authors:** Lambert T. Leong, Michael C. Wong, Yong E. Liu, Yannik Glaser, Brandon K. Quon, Nisa N. Kelly, Devon Cataldi, Peter Sadowski, Steven B. Heymsfield, John A. Shepherd

**Affiliations:** 1 Molecular Bioscience and Bioengineering at University of Hawaii, Honolulu, HI USA; 2 Department of Epidemiology, University of Hawaii Cancer Center, Honolulu, HI USA; 3 Information and Computer Science at University of Hawaii, Honolulu, HI USA; 4 Pennington Biomedical Research Center, Louisiana State University, Baton Rouge, LO USA

**Keywords:** Epidemiology, Whole body imaging

## Abstract

****Background**:**

Body shape, an intuitive health indicator, is deterministically driven by body composition. We developed and validated a deep learning model that generates accurate dual-energy X-ray absorptiometry (DXA) scans from three-dimensional optical body scans (3DO), enabling compositional analysis of the whole body and specified subregions. Previous works on generative medical imaging models lack quantitative validation and only report quality metrics.

****Methods**:**

Our model was self-supervised pretrained on two large clinical DXA datasets and fine-tuned using the Shape Up! Adults study dataset. Model-predicted scans from a holdout test set were evaluated using clinical commercial DXA software for compositional accuracy.

****Results**:**

Predicted DXA scans achieve *R*^2^ of 0.73, 0.89, and 0.99 and RMSEs of 5.32, 6.56, and 4.15 kg for total fat mass (FM), fat-free mass (FFM), and total mass, respectively. Custom subregion analysis results in *R*^2^s of 0.70–0.89 for left and right thigh composition. We demonstrate the ability of models to produce quantitatively accurate visualizations of soft tissue and bone, confirming a strong relationship between body shape and composition.

****Conclusions**:**

This work highlights the potential of generative models in medical imaging and reinforces the importance of quantitative validation for assessing their clinical utility.

## Plain language summary

Body composition, measured quantities of muscle, fat, and bone, is typically assessed through dual energy X-ray absorptiometry (DXA) scans, which requires specialized equipment, trained technicians and involves exposure to radiation. Exterior body shape is dependent on body composition and recent technological advances have made three-dimensional (3D) scanning for body shape accessible and virtually ubiquitous. We developed a model which uses 3D body surface scan inputs to generate DXA scans. When analyzed with commercial software that is used clinically, our model generated images yielded accurate quantities of fat, lean, and bone. Our work highlights the strong relationship between exterior body shape and interior composition. Moreover, it suggests that with enhanced accuracy, such medical imaging models could be more widely adopted in clinical care, making the analysis of body composition more accessible and easier to obtain.

## Abstract (web summary)

Leong et al. develop a generative deep learning model that produces analyzable dual energy X-ray absorptiometry (DXA) scans from 3D body surface scans. Model-generated DXA scans are analyzed using clinical commercial software and yield accurate quantities of fat, lean, and bone mass for total body and body subregions.

## Introduction

Body composition is indicative of many disease states and adverse health outcomes^1^. For example, obesity and sarcopenic obesity (high adiposity) are associated with cardiovascular disease and diabetes^2^, and sarcopenia and frailty (loss of lean mass and muscle)^3^ are associated with increased mortality^4,5^. In addition to total or whole-body (WB) composition, specific subregional composition has also been shown to have strong and unique associations to specific health outcomes^6^. For instance, every kilogram increase in appendicular lean mass (ALM) was shown to be associated with about a 10% reduction in mortality in elderly individuals^7^. However, with limited exceptions, only body composition assessments derived from advanced imaging methods can effectively segment the body to quantify appendicular regions. Commonly used anatomical cut points or subregions from dual-photon absorptiometry^8,9^ and then dual-energy X-ray absorptiometry [DXA^10,11^] whole body images were adopted in the 1980s and they have since been incorporated into standard clinical practice with little to no modification to original subregion definitions. Some relevant examples of standard DXA subregion for body composition include ALM’s association to frailty^12^, visceral adipose tissue (VAT) and subcutaneous adipose tissue of the trunk region being associated to cardiometabolic outcomes^13^, leg fat and lean mass in diabetes patients^14^, leg fat mass (FM) and fat-free mass (FFM) being associated to frailty and injury recovery^15^. Besides these historical subregions, DXA offers the capability to explore body composition within user-defined subregions and some examples include user-defined abdominal subregions to monitor liver iron concentration^16^ and leg subregions to monitor injury recovery^17^.

While DXA is considered a criterion method for acquiring body composition, exposure to ionizing radiation limits accessibility and frequent use in individuals. Specially trained and licensed technicians are often required to operate DXA systems and mitigation of dose accumulation. Computed tomography (CT)^18^ and magnetic resonance imaging (MRI) are alternatives to DXA and also offer regional body composition measures^19^. However, the limitations that hinder DXA accessibility and broader use are not overcome by either method. Highly skilled technicians are still needed to operate these systems, they are expensive for the user and facility to maintain, and CT utilizes even higher ionizing radiation doses than DXA. Bioelectrical impedance analysis (BIA) is a common non-image-based and accessible body composition method that can segment the body into trunk, arms and legs using selective placement of up to eight electrodes^20^. However the division between subregions is only vaguely definable, dependent on the composition distribution of the individual. DXA, CT, and MRI have precisely defined anatomical cut points verifiable from the image. Thus, neither DXA, CT, MRI, or BIA are ideal methodologies for the frequent monitoring of common or user-defined subregions of body composition with metabolic significance.

An unlikely candidate, three-dimensional optical surface scanning (3DO), has demonstrated the ability to accurately and precisely measure total and regional body composition by way of detailed modeling of body shape^21,22^. Body shape is deterministically driven by the internal distributions of fat and muscle soft tissue. Body shape has been shown to have associations with blood metabolites, strength^23^, and metabolic syndrome^24^ demonstrating the broad health utility of 3DO technology. Recent advances in depth camera technology has made whole-body scanning inexpensive and fast. These systems are broadly used for monitoring body shape and composition in homes, recreational facilities, and clinical settings^25,26^. 3DO depth cameras are so ubiquitous that they can be found in many laptop computers, cell phones and gaming systems. Advances in image processing and machine learning techniques have resulted in body shape models that accurately predict body composition from 3DO scans^22,27,28^. A drawback to statistical and machine learning shape models for composition is that these models typically predict singular scalar values per each body composition measurement. Exploration into additional hypotheses is not possible with such models and requires retraining. Like other mentioned image-based methods, previously published body shape models have not been flexible enough to allow for ad hoc user-defined subregional analysis. Adding ad hoc user-based analysis for body composition to 3DO whole body scans would satisfy the ideal conditions outlined above.

We present a novel approach, a cross-modality image-to-image model for quantitative body composition image predictions from 3DO, to the best of our knowledge. We use a generative deep-learning model that maps 3DO to DXA scans. Our model, Pseudo-DXA, outputs DXA scans in format usable by a commercially available body composition analysis software so that this advancement can be readily used by clinicians and researchers. Further, using this approach allows for direct validation of user-defined regions using paired DXA and 3DO scans. We further show that the Pseudo-DXA body composition results are surrogate measures to DXA by comparing DXA and Pseudo-DXA to metabolic blood markers. Pseudo-DXA was only achievable due to 1) the availability of large datasets, over 1000 sets, that included matched 3DO and DXA, 2) advances in deep learning and self-supervised training methods, and 3) technological advances which lead to improved 3DO capture and processing power needed to train our final model.

## Methods

The development of our Pseudo-DXA model consisted of two distinct phases; a self-supervised learning (SSL) pretraining phase and a cross-modality fine-tuning phase. Pretraining strategies are commonly used in deep learning to increase robustness and combat overfitting when dataset sizes are modest. Imaging models have shown improvements in performance on downstream task^29,30^ as a result of effective pretraining. A SSL^31–33^ training strategy was employed which enabled the model to utilize large datasets of unlabeled DXA scans to learn the important and complex imaging features needed for generating accurate scans. Once the model learned to generate DXA scans during pretraining, it was then tuned specifically to learn the mapping between 3DO and DXA scans. The following sections detail the development in full.

### Study populations

The SSL pretraining phase utilized DXA data from two studies, Health, Aging, and Body Composition (Health ABC)^34,35^ and Bone Mineral Density in Childhood Study (BMDCS)^36^. The Health ABC study is a prospective cohort study of 3075 individuals (48.4% male, 51.6% female) aged 70–79 years at the time of recruitment, 41.6% of whom are Black with the remaining 58.4% being non-Hispanic White. Participants were recruited from Medicare-eligible adults in metropolitan areas surrounding Pittsburgh, Pennsylvania and Memphis, Tennessee and were monitored yearly for 10 years. The BMDCS is also a prospective study cohort of 2014 individuals (49.3% male, 50.76% female) aged 5–20 years. Participants were recruited at five clinical centers in the US and participants were followed for 6 years which included annual assessments. Although both the Health ABC and BMDCS studies were longitudinal, we utilized the data in a cross-sectional manner for the SSL phase.

The cross-modality training phase utilized 3DO scans and DXA scans from a third study, Shape Up! Adults (SUA (NIH R01 DK109008))^23^. This study is a cross-sectional study of healthy adults. Participants were recruited at Pennington Biomedical Research Center (PBRC), University of Hawaii Cancer Center (UHCC), and University of California, San Francisco (UCSF). Recruitment was designed to result in a diverse population that is well stratified by sex, age, ethnicity, and body mass index (BMI). Patient demographics for all phases are shown in Table 1 and a flowchart detailing the data sources for each training phase is shown in Supplementary Fig. 1. For this study, all participants signed an informed written consent form which was approved by each respective study institutional review boards (IRB). The Heath ABC protocol was approved by the IRB at each field center (University of Pittsburg, PA and University of Tennessee, Memphis, TN), the BMDCS protocol was approved by the IRB at each clinical center (The Children’s Hospital of Philadelphia, Cincinnati Children’s Hospital Medical Center, Creighton University, Children’s Hospital Los Angeles, and Columbia University) and the data coordinating center (Clinical Trials and Survey Corporation). The Shape Up! Adults protocol, which covers this study, was approved by the IRBs at PBRC, UCSF, and the University of Hawaii Office of Research Compliance.

**Table 1:** Datasets and demographics

		Self-Supervised Learning		Supervised Learning
Cohort		Health ABC	BMDCS	Shape Up! Adults
	*N*	17541	8065	714
Sex				
	Male, Female	8419, 9122 (48%, 52%)	3929, 4136 (49%, 51%)	344, 370 (48%, 52)
Age (years)				
	Mean (SD)	74.1 (2.87)	13.61 (4.17)	43.99 (15.88)
	Median [Min, Max]	73 [68, 80]	13.3 [5.00, 23.4]	42.12 [18.00, 89.00]
Ethnicity				
	American Indian	0	216 (3%)	0
	Asian	0	556 (7%)	148 (21%)
	Black	7542 (43%)	2101 (26%)	144 (20%)
	Hispanic	0	1243 (15%)	89 (13%)
	NHOPI	0	22 (0.28%)	46 (6%)
	White	9999 (57%)	3927 (49%)	287 (40%)
Height (cm)				
	Mean (SD)	166.23 (9.37)	154.09 (18.17)	167.48 (17.51)
	Median [Min, Max]	165.86 [136.50, 200.70]	157.47 [104.90, 190.03]	166.33 [149,192]
Weight (kg)				
	Mean (SD)	75.81 (15.05)	50.25 (18.04)	85.31 (15.76)
	Median [Min, Max]	75.20 [33.5, 141.00]	50.5 [17, 111.2]	81.05 [60.1, 143]
BMI				
	Mean (SD)	27.39 (4.82)	20 (4.00)	31.58 (6.8)
	Median [Min, Max]	26.87 [14.59, 51.99]	19.85 [13.4, 38.5]	30.5 [19.0, 52]

### DXA data

All DXA scans were acquired on Hologic (Hologic Inc., MA, USA) scanners of similar models. The Health ABC whole-body scans utilized were collected using Hologic QDR 4500 systems and attempts were made to collect DXA scans on eight occasions throughout the study. Hologic QDR4500, Delphi, and Discovery models were used to acquire whole-body DXA scans for the BMDCS and scans were acquired yearly for 6 years. Participants of the Shape Up! Adults study received whole-body DXA scans with a Hologic Discovery/A system. Some participants also received duplicate precision scans within the same visit. To estimate test–retest precision, Shape Up participants were scanned twice with repositioning between the scans. Height and weight measures were available for all participants. Manufacturer-defined acquisition protocols were used to ensure reproducibility and standardization of patient positioning^37,38^. For each participant, the raw dual-energy attenuation images with their respective calibration images were represented at a bit depth of 16-bit.

### 3DO scan data

The 3DO scans were acquired on Fit3D Proscanner (Fit3D Inc., CA, USA). Participants were required to wear form-fitting tights, a swim cap, and sports bras if female. Participants grasped telescoping handles on the scanner platform and stood upright with arms positioned straight and abducted from their torso while the scan platform made one revolution. Final point clouds were converted to a mesh connected by triangles with ~300,000 vertices and 60,000 faces. Scans were then standardized to the same T-pose, same coordinates system, and same 110 K vertices using Meshcapade’s (Meshcapade GmbH, Tübingen, Germany) skinned multi-person linear model (SMPL) service^39^.

### Deep learning modeling

In an attempt to mitigate overfitting, data sets for both training phases were split into train, validation, and holdout test sets using 80%, 10%, and 10% split, respectively. These split ratios applied to both the SSL pretraining phase and the Pseudo-DXA supervised training phase. The data was split based on participant subject ID to ensure that all duplicate scans remained together in the train, validation, or test splits. Splits were also performed in a stratified fashion to best preserve the age, height, weight, and BMI distributions within each data subset.

### Pretraining self-supervised learning

Pretraining via SSL allowed us to leverage the large set of raw DXA data from the BMDCS and Health ABC studies. A variation auto-encoder (VAE)^40^ network architecture was chosen for its modular nature. VAEs consist of two main subnetwork components which include an encoder and a generator. In brief, the encoder portion of the network is tasked with learning the important imaging information from the DXA scans and encoding them into a reduced number of features known as a latent space. The generator is tasked with generating the original image from the reduced features or latent space.

Our encoder network was made up of Densenet121^41^ and the generator consisted of consecutive two-by-two bilinear up sampling and 2D convolutional units modeled after the Super resolution networks^42^ architecture. Inputs were the DXA images and VAE output predictions of the original reconstructed DXA scan. A VGG-16 perceptual loss^43^ and a custom DXA content loss function^44^ was used to compare the predicted image to the original DXA input. Image inputs were augmented with a combination of translation and rotation operations to de-incentivize the network from memorizing the data. Destructive augmentations were also used during training. Portions of the input image were randomly scrambled and noise was added to force the network to use the surrounding image structure to in paint^45^ the destroyed regions. Hyperparameters which include learning rate, learning rate decay, and batch size were tuned using an automated Python module entitled Sherpa^46^. An early stopping parameter halted training when the validation loss ceased to decrease significantly. The holdout test set was used to evaluate the VAE-predicted images. If the VAE was able to produce images with minimal error, we assumed that it has effectively learned the DXA image data type. The weights of the trained VAE were frozen.

### Pseudo-DXA modeling

The trained VAE generator subnetwork provided the starting point for final 3DO to DXA model. A Pointnet^47^ model was attached to the VAE generator and was used to map the 3DO scans into the DXA space, see Supplementary Fig. 2. Due to computational constraints, a preprocessing step was applied, on the fly, to reduce the 110 K vertices 3DO scans to 20% of the full resolution. Sherpa was again used during the construction of the final Pseudo-DXA model to optimize hyperparameters. Early stopping was used to determine when to halt training after which final evaluation was performed on the holdout test set.

### Image quality analysis

Normalized means absolute error (NMAE), peak signal-to-noise ratio (PSNR), and structural similarity index (SSIM) are common computer vision image quality metrics and were computed for each test set observation^48,49^. NMAE values range between 0 and 1 where a lower value indicates less error and zero indicates a perfect reconstruction. NMAE is not invariant to positioning differences and thus we also use PSNR and SSIM which are less prone to error introduced by positioning. Higher PSNR values are ideal and for the 16-bit DXA images, 20 dB and higher are considered acceptable. SSIM ranges between 0 and 1 where higher values indicate better image quality and 1 indicates a perfect reconstruction.

### Body composition analysis

Quantitative image analysis was performed in addition to evaluations with standard image quality metrics. Hologic, Inc. Apex version 5.5 software was used to derive body composition measures from both the actual and Pseudo-DXA scans with the NHANES option disabled. An example of a Pseudo-DXA scan analysis is shown in Supplementary Fig. 3. The red lines indicate predefined regions of interest (ROI) that are essential to computing body composition. Although we used the “Auto-Analyze” feature, scans require manual review to ensure the regions are placed correctly. Also, this software is intended for clinical use and not designed for high throughput analysis and, therefore, was a consideration when determining the size of our final holdout test set.

### Special subregional composition analysis

To further demonstrate the validity and utility of Pseudo-DXA scans, we performed analysis on user defined or special subregions. The two subregions used for this analysis are shown in Supplementary Fig. 6 where R1 is the right thigh ROI and R2 is the left thigh ROI. The ROIs for both thigh subregions were defined similar to lower-body segmental analysis using DXA performed by Hart et al.^17^. The tops of each ROI were aligned with the patient’s iliac crest while the bottom of the ROI was aligned with the space between the patient’s femur and tibia. Each ROI was also aligned such that the medial angled edge of the ROI touched the anterior superior iliac spine and pubic arc, see Supplementary Fig. 6. All singleton participant actual DXA and pseudo-DXA scans were analyzed in this fashion to obtain subregion-specific composition.

### Statistical analysis

Regression analysis was used to evaluate the agreement of body composition between Pseudo-DXA images and actual DXA images. FM, FFM, and bone mass were evaluated for the entire body as well as subregions which include the trunk, arms, and legs. The coefficients of determination (*R*^2^) and root mean squared error (RMSE) were reported for all body composition comparisons. Scale weight was evaluated as a covariate and the adjusted *R*^2^ and RMSE values were computed.

Select participants received duplicate 3DO and DXA scans. Coefficients of variation (%CV) and root mean squared standard error (RMSE(CV)) were calculated to quantify test–retest or short-term precision^50^ of both the Pseudo-DXA model and DXA model. Precision is evaluated with respect to fat, lean, and bone mass for the entire body as well as subregions, which includes the trunk, arms, and legs.

### Reporting summary

Further information on research design is available in the Nature Portfolio Reporting Summary linked to this article.

## Results

The SSL data set consisted of 25,606 (48% male and 52% female) total scans from both the Health ABC and BMDCS studies, see Table 1. Eight hundred and eleven DXA scans were excluded from the Health ABC study because they were not acquired on a Hologic QDR 4500 or later system, resulting in a different raw image format. Scans were also excluded based on size and the height and width were exactly 150 and 109 pixels, respectively. Forty-eight Health ABC scans and 2812 BMDCS scans were excluded from SSL.

At the time of this analysis, a total of 714 participants received both a 3DO and DXA scan on the same day as a part of the Shape Up! Adult, see Table 1. Select participants received duplicate scans on both the 3DO and DXA systems for precision monitoring and this resulted in 1169 pairs of scans. The paired data set has a holdout test set of size 70 unique individuals of which 50 participants received duplicate DXA and 3DO scans. All the following results are reported on the 70 unique participants that the Pseudo-DXA model had not seen during training.

### Image quality assessment of 3DO to DXA model

**Fig. 1:**
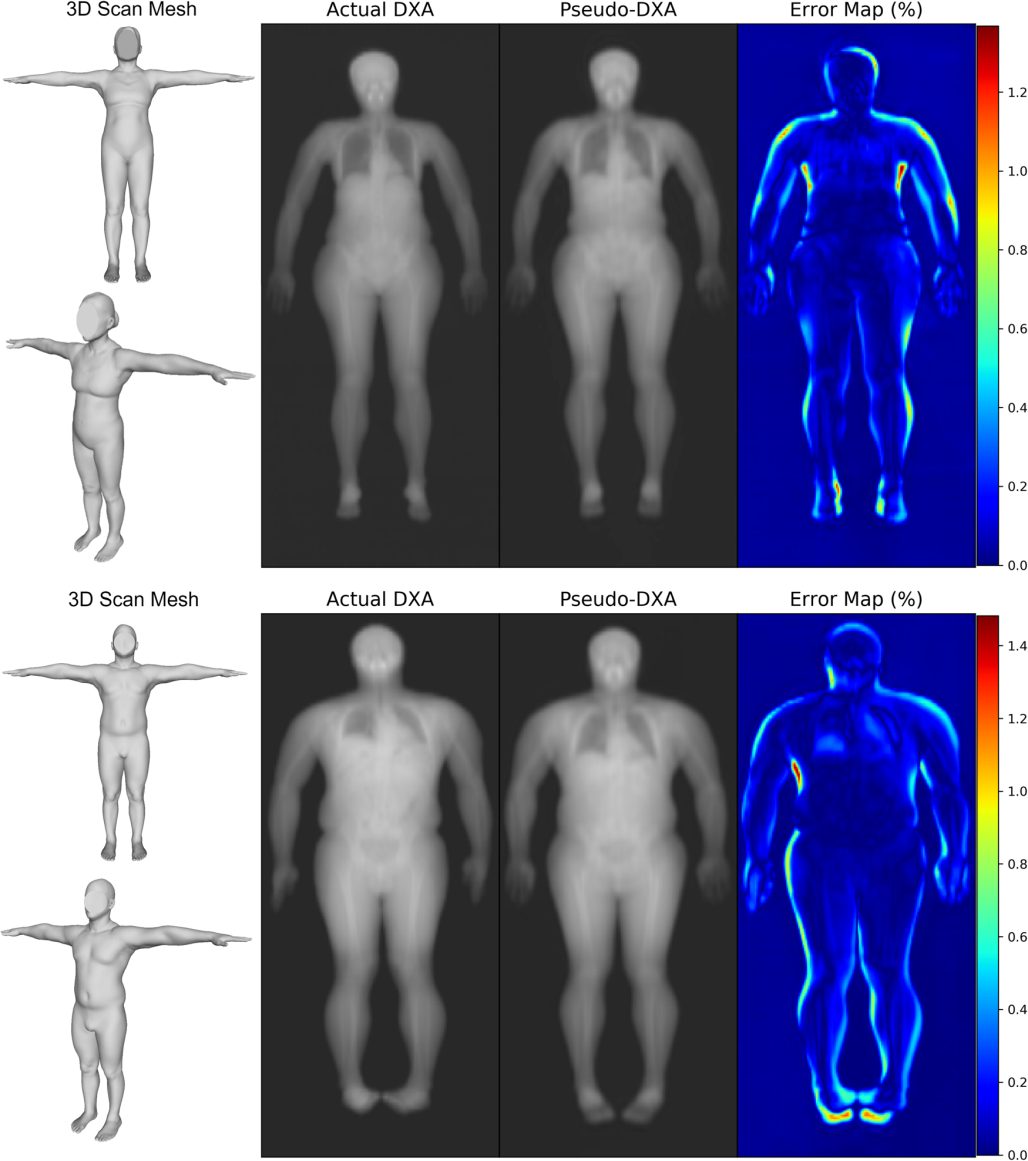
Female (top) and male (bottom) test set examples of model inputs and prediction comparisons. Two views of a participant 3D scan standardized to the T-pose, the actual DXA scan, the Pseudo-DXA model predicted scan, and the error map comparing the actual DXA to the Pseudo-DXA. Error maps represent percent error where zero and 100 equate to no error and the maximum error, respectively.

The NMAE, PSNR, and SSIM were computed and the average values from all predicted images 0.15, 38.15 dB, and 0.97, respectively. Good quality 16-bit images have low NMAE near zero, PSNR values greater than 25, and high SSIM values near one^48,49^. Ideal ranges and reference values are shown in Table 2 Some predictions resulted in a high NMAE with the highest value being 0.38. NMAE is not invariant to position, and positioning differences can lead to worse NMAE metrics, while PSNR and SSIM may show little to no change. Figure 1 contains scans from a representative female and male example within the holdout test set. Error maps show the majority of the errors around the skin edges and feet which suggest that positioning differences are the main source of pixel differences.

**Table 2:** Pseudo-DXA image quality performance.

*N* = 70	Ideal reference value [Range]	Mean (SD)	Minimum	Maximum
NMAE ↓	0 [0,1]	0.15 (0.06)	0.06	0.39
PSNR ↑	>25 dB [0, INF]	38.15 (2.7)	31.70	43.88
SSIM ↑	1 [0,1]	0.97 (0.03)	0.89	0.99

Normalized mean absolute error (NMAE), peak signal-to-noise ratio (PSNR), and structure similarity index (SSIM) were computed for all holdout test set predictions. The means, standard deviations (SD), minimum, and maximum are reported.

### Pseudo-DXA quantitative analysis for body composition

Comparing Pseudo-DXA and actual scans (Table 3) resulted in *R*^2^ values for whole-body FM, lean soft tissue or FFM, bone mineral content (BMC), and total mass of 0.66, 0.82, 0.72, and 0.89, respectively. RMSEs for whole body FM, FFM, BMC, and mass are 6.89, 7.66, 0.30, and 5.48 kg, respectively. Standard DXA analysis also reports composition for predefined subregions which include the trunk, arms, and legs. Comparisons of Trunk FM and FFM resulted in *R*^2^ of 0.71 and 0.81, respectively; arm FM, FFM, and BMC resulted in *R*^2^ of 0.60, 0.84, and 0.71, respectively, and leg FM, FFM, and BMC resulted in R^2^ of 0.48, 0.83, and 0.80, respectively.

**Table 3:** Evaluation of pseudo-DXA images for quantitative accuracy.

			Raw		Weight Corrected	
*N* = 70	Measurement	Units	*R*^2^	RMSE	*R*^2^	RMSE
Whole Body	Total Mass	kg	0.89	5.48	0.99	4.15
	FM	kg	0.66	6.89	0.73	5.32
	FFM	kg	0.82	7.66	0.90	6.56
	BMC	kg	0.72	0.30	0.74	0.24
	BMD	g/cm^3^	0.50	0.12	0.53	0.12
	VAT	kg	0.52	0.22	0.56	0.13
Predefined Subregions	Trunk FM	kg	0.71	3.25	0.80	2.39
	Trunk FFM	kg	0.81	4.35	0.89	3.09
	Arm FM	kg	0.60	0.56	0.67	0.34
	Arm FFM	kg	0.84	0.52	0.85	0.54
	Arm BMC	kg	0.71	0.03	0.74	0.03
	Arm BMD	g/cm^3^	0.70	0.07	0.74	0.12
	Leg FM	kg	0.48	1.62	0.56	0.83
	Leg FFM	kg	0.83	1.38	0.87	1.19
	Leg BMC	kg	0.80	0.05	0.84	0.05
	Leg BMD	g/cm^3^	0.66	0.11	0.72	0.14

Mass, fat (FM), lean or fat-free mass (FFM), visceral adipose tissue (VAT), bone mineral density (BMD), and bone mineral content (BMC) from the whole body and subregions were measured on actual and Pseudo-DXA scans. Univariate regression analysis was used to compare predicted and actual values. Coefficients of determination (*R*^2^) and root mean squared errors (RMSE) are reported. In addtion to the raw values, we report results when using scale weight to correct body composition values.

For DXA, attenuation is directly related to the mass of the object within the X-ray path. Since the Pseudo-DXA model was not well calibrated specifically to account for this relationship, we use scale weight to correct the derived body composition. When correcting with scale weight, *R*^2^ values for whole-body FM, FFM, BMC, and total mass of 0.73, 0.90, 0.74, and 0.99, respectively. Weight corrections improved RMSEs to 5.32, 6.56, 0.24, and 4.15 kg for FM, FFM, BMC, and total mass, respectively.

Raw and weight-corrected Bland-Altman plots for each corresponding whole body and predefined subregion composition are shown in Supplementary Fig. 4 and Supplementary Fig. 5, respectively. There appears to be no obvious positive or negative trend, and the scatter is spread evenly.

### Special subregional performance

Subregional analysis was performed on 70 participants from the holdout test set, see Table 4. If participants received two DXA scans, the first scan was used for the analysis. The *R*^2^s for FM, FFM, and total mass of the right leg were 0.72, 0.77, and 0.90, respectively and the RMSEs were 1.34, 1.27, and 0.72 kg, respectively. The *R*^2^s of FM, FFM, and total mass of left leg were 0.70, 0.78, and 0.89, respectively, and the RMSEs were 1.25, 1.26, and 0.71 kg, respectively.

**Table 4:** Composition of special subregional analysis.

*N* = 70		Left Leg		Right Leg	
Measurement	Units	*R*^2^	RMSE	*R*^2^	RMSE
FM	kg	0.70	1.25	0.72	1.34
FFM	kg	0.78	1.26	0.77	1.27
Total Mass	kg	0.89	0.71	0.90	0.72

Fat mass (FM), fat-free mass (FFM), and total mass are evaluated for left and right leg special subregions. Coefficients of determination(*R*^2^) and root mean squared errors (RMSE) are reported.

Bland-Altman plots for each left and right leg FM, FFM, and total mass are shown in Supplementary Fig. 7. There appears to be no obvious positive or negative trend, and the scatter is spread evenly.

### Test–retest precision analysis

Fifty participants within the holdout test set received duplicate 3DO and DXA scans. These duplicates allowed us to evaluate the precision of our model against the actual DXA system. Test–retest precision for both DXA and Pseudo-DXA scans was assessed for all whole body and standard subregional DXA body composition measures, and the results are presented in Table 5. Precision CV ranged between 0.21–7.04% for DXA and 0.15–6.67 for Pseudo-DXA. Precision for both DXA and Pseudo-DXA were comparable; *p* values above 0.05. Pseudo-DXA demonstrated better precision than DXA on whole body measures of mass, bone mineral density, and VAT with %CV of 0.15, 0.36, and 6.67, respectively, compared to 0.21, 0.43, and 7.04, respectively for DXA. Pseudo-DXA had better precision for the subregional measure of trunk FFM with a %CV of 0.79 compared to 0.80 for DXA.

**Table 5:** DXA vs pseudo-DXA Test–retest precision.

*N* = 50			DXA		Pseudo-DXA	
	Measurement	Units	CV	RMSE (CV)	CV	RMSE (CV)
Whole Body	Total Mass	kg	0.21	0.16	0.15	0.11
	FM	kg	0.57	0.12	0.59	0.17
	FFM	kg	0.34	0.18	0.40	0.18
	BMC	kg	0.49	0.01	0.59	0.01
	BMD	g/cm^3^	0.43	0.47	0.36	0.36
	VAT	kg	7.04	0.04	6.67	0.03
Predefined Subregions	Trunk FM	kg	1.26	0.13	1.44	0.20
	Trunk FFM	kg	0.80	0.21	0.79	0.17
	Arm FM	kg	2.75	0.04	5.05	0.09
	Arm FFM	kg	1.32	0.04	3.89	0.10
	Arm BMC	kg	1.14	0.00	2.81	0.00
	Arm BMD	g/cm^3^	0.81	0.60	1.08	0.83
	Leg FM	kg	1.54	0.06	3.96	0.21
	Leg FFM	kg	1.14	0.10	2.70	0.20
	Leg BMC	kg	0.84	0.00	1.51	0.01
	Leg BMD	g/cm^3^	0.72	0.82	0.74	0.79

Fitfty participants in the holdout test set received duplicate DXA and 3D scans. The test and retest scan values for each participant were used to compute the percent coefficient of variation (%CV) and root mean square error (RMSE) precision metrics. Precision metrics were computed for mass, fat (FM), lean or fat free mass (FFM), visceral adipose tissue (VAT), bone mineral density (BMD) and bone mineral content (BMC) from the whole body and subregions on actual and Pseudo-DXA scans.

## Discussion

We present the Pseudo-DXA model which has successfully learned to predict interior body composition from exterior body shape. From a 3DO scans, Pseudo-DXA generates a DXA scan of high image quality that can be quantitatively analyzed using standard body composition software. Our experiments confirm that soft tissue distribution and boney structure play an important role in determining a unique exterior body shape. While previous work has shown that body shape is predictive of aggregate body composition values^21,23^, this work extracts a much richer feature set from 3DO body scans than previous studies. In fact, body composition values reported in previous works can be derived from images output from our Pseudo-DXA model.

Pseudo-DXA demonstrated similar if not indistinguishable test–retest precision for DXA measurements when compared to the original DXA images. With similar precision and no ionizing radiation, 3DO may be used more frequently than DXA to obtain a higher fidelity to change in body composition than DXA. As outlined in Gluer et al^50^., four measures at baseline and follow-up visits reduces the precision error by 2 times and thus shorten the monitoring time interval by half^51^.

To our knowledge, this work is the first instance in which deep learning reconstructed images were shown to be compatible with a clinical medical imaging algorithm and achieve quantitatively accurate results. Other noteworthy medical image reconstruction models, such as RegGAN for MRI^52,53^ or Shan’s for low-dose CT^54^, only report aggregate image quality metrics^55^ which has limited clinical utility. Achieving quantitatively accurate image reconstructions is more difficult since errors in the magnitude or relative pixel values, not discernable by eye, can render the images useless for quantitative measures. Attempts at quantitative accuracy were made by Wang et al. using body shape to create CT abdominal images to quantify visceral adipose tissue^56^ and liver steatosis^57^. However, Wang used the CT scans themselves as the shape which is perfectly registered with the target CT scans. Although this shows feasibility of their approach, much effort is still needed to show that 3DO body shape would accurately predict the same measures. In our work, we used 3DO scans of standing patients to predict the supine dual-energy X-ray images. Unlike their work, our work could not benefit by the spatial registration of the body.

This work is not without limitations. While our model performed well when predicting whole body and subregional bone measurements, predictions were derived from the external body shape which is dominated by fat and muscle distribution. It is reasonable to think that body shape would be highly correlated to bone density especially for cortical bone since it makes up 80% of bone mass and has a very slow annual turnover^58^. Thus, Pseudo-dxa models may be good estimates of what the bone mass should be for given the muscle fat distribution but not a good indicator of higher turnover diseases that impact trabecular bone. Further, the model may be impacted by pathologies related to tumors and artifacts related to arthroplasty since it is unclear how various pathologies manifest as 3DO body shape signals, if at all. Pseudo-DXA images underperformed on some of the DXA compositional values, mainly associated to measure of fat. The data set size of our paired 3DO and DXA was a limitation. However, we utilized self-supervised learning on DXA images to address a portion of this issue. Pretraining the 3DO portion of the Pseudo-DXA model would likely benefit overall performance and can be performed with large unpaired 3DO datasets^59,60^. Lastly, underperformance of our model could be attributed to differences in demographic distributions within the datasets. The self-supervised learning dataset consisted of two cohorts of which one was young and the other older with median ages of 13.3 and 73, respectively. The median age of the supervised learning cohort was 42.1. Although the age distribution of the supervised learning cohort overlapped with the other cohorts, it is a potential source of unavoidable bias that we acknowledge as a limitation.

We conclude that 3DO scanning can provide access to an abundance of information beyond current clinical tools being used in obesity reduction. Our Pseudo-DXA model is end-to-end meaning it can take a 3DO scan and produce an image that can be analyzed for clinical measures of composition. The relationship between body composition and shape learned by our model demonstrates clinical relevance and warrants further research into 3DO body shape as an indicator of health. It is important to note that this work is not meant to demonstrate a replacement for DXA body composition but rather demonstrate translational health and medical applications of the information afford from accessible 3DO scans. Lastly, when possible, future medical image reconstruction deep learning work should be held to the standard of performing quantitative analysis as it will improve clinical translation^61^.

### Supplementary information

Supplementary Information

Reporting Summary

## Data Availability

The Health ABC data are available from the National Institute on Aging, but restrictions apply to the availability of these data, which were used under license for the current study, and so are not freely available. Data, however can be requested through the study’s website at healthabc.nia.nih.gov. The BMDCS data can be accessed through the NICHD DASH website (https://dash.nichd.nih.gov/). The Shape Up! Adults data are available from the corresponding author upon reasonable request or at https://shapeup.shepherdresearchlab.org/for-researchers/.

## Code availability

Code for building and training our final 3D to analyzable DXA model can be found at https://github.com/LambertLeong/Pseudo-DXA^62^, 10.5281/zenodo.10183202.
