# Supplementary material for: Generative deep learning furthers the understanding of local distributions of fat and muscle on body shape and health using 3D surface scans
Source: Commun Med (Lond). 2024 Jan 30;4:13. doi: 10.1038/s43856-024-00434-w (PMC10824755; doi:10.1038/s43856-024-00434-w)
Supplement: Supplementary file 1 — Supplementary Information [file 43856_2024_434_MOESM1_ESM.pdf]

SUPPLEMENTARY MATERIAL

Deep learning furthers the understanding of local distributions of fat and muscle on body shape and health using 3D surface scans

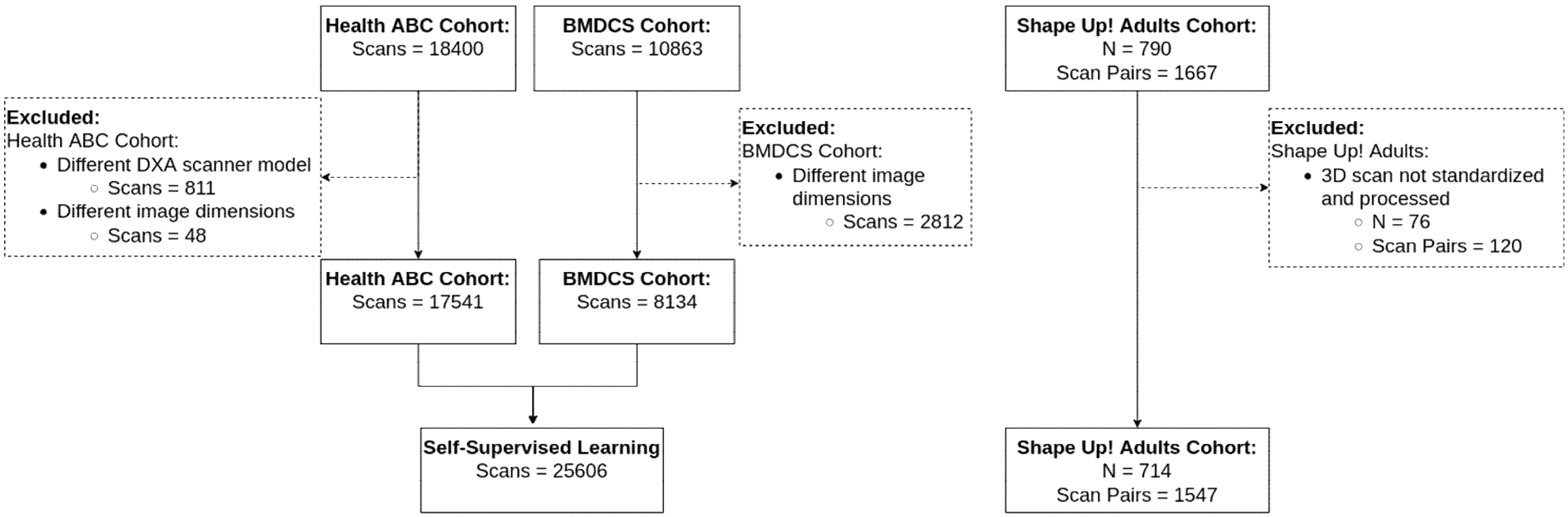

Supplemental Figure 1. Flowchart of participants and data used in self-supervised learning, left, and the Pseudo-DXA training, right.

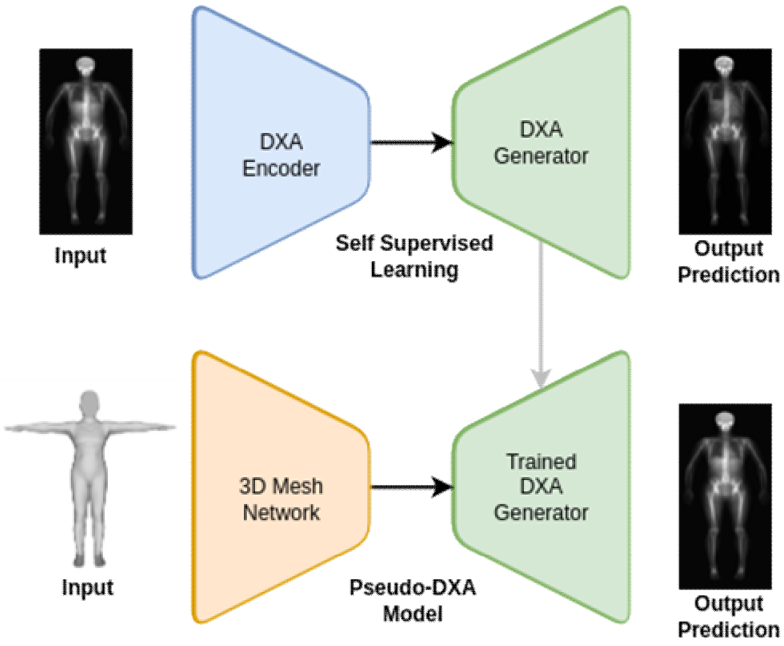

Supplemental Figure 2. Training Scheme and Network Architecture. A Variational auto-encoder (VAE) trained on unpaired DXA data in a self-supervised fashion to learn to reconstruct accurate DXA images (top). A 3D mesh network is attached to the trained VAE generator to create the Pseudo-DXA model (bottom).

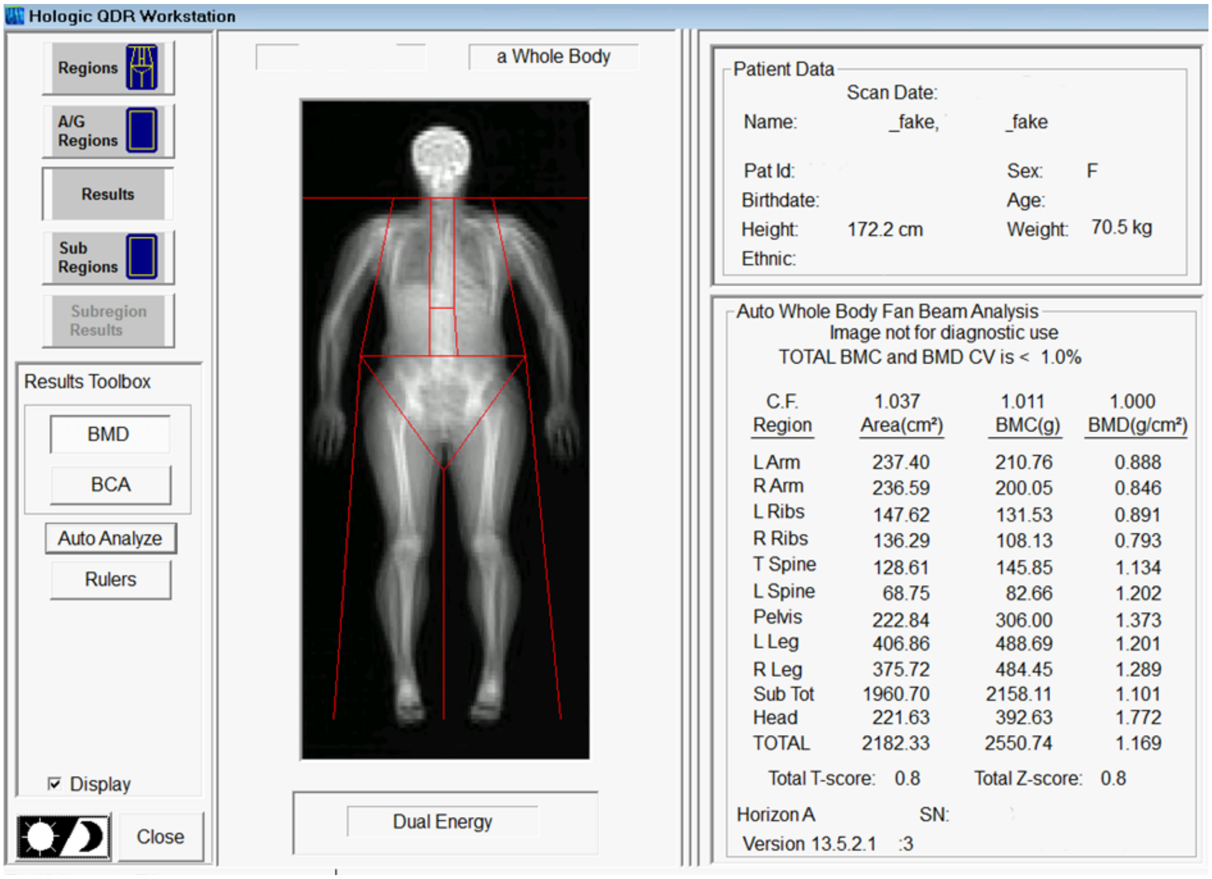

Supplemental Figure 3. Example Quantitiative Analysis of Pseudo-DXA Scan Using Clinical Software. Pseudo-DXA scans were analyzed using Hologic incs Apex 5.5 software. Anatomical land marks are used to place red lines indicating regions of interest from which subregional body composition is computed from.

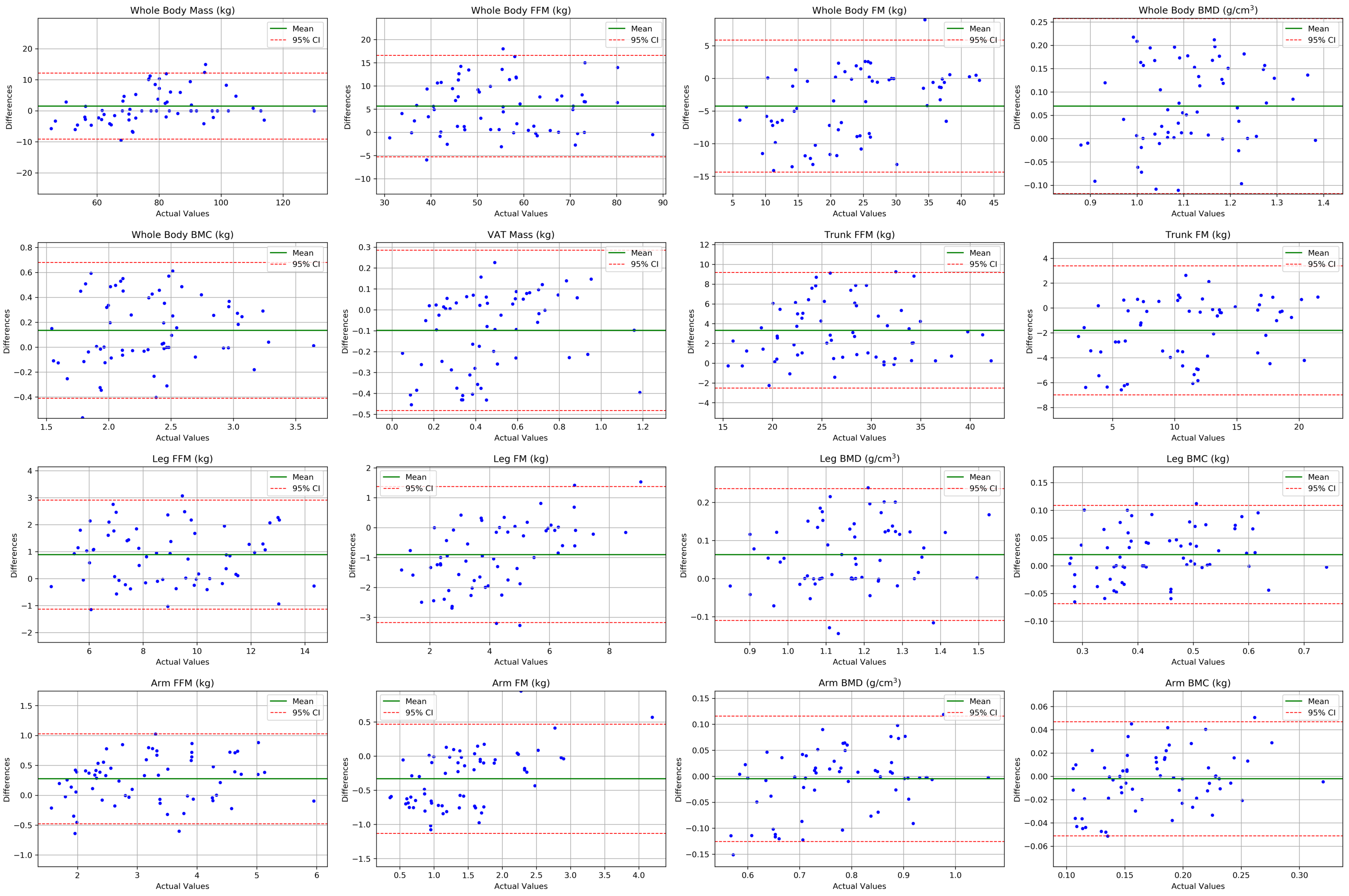

Supplemental Figure 4 Bland-Altman plots for actual DXA vs Pseudo-DXA measurements of fat mas (FM), fat free mass (FFM), bone mineral content (BMC) and bone mineral density (BMD).

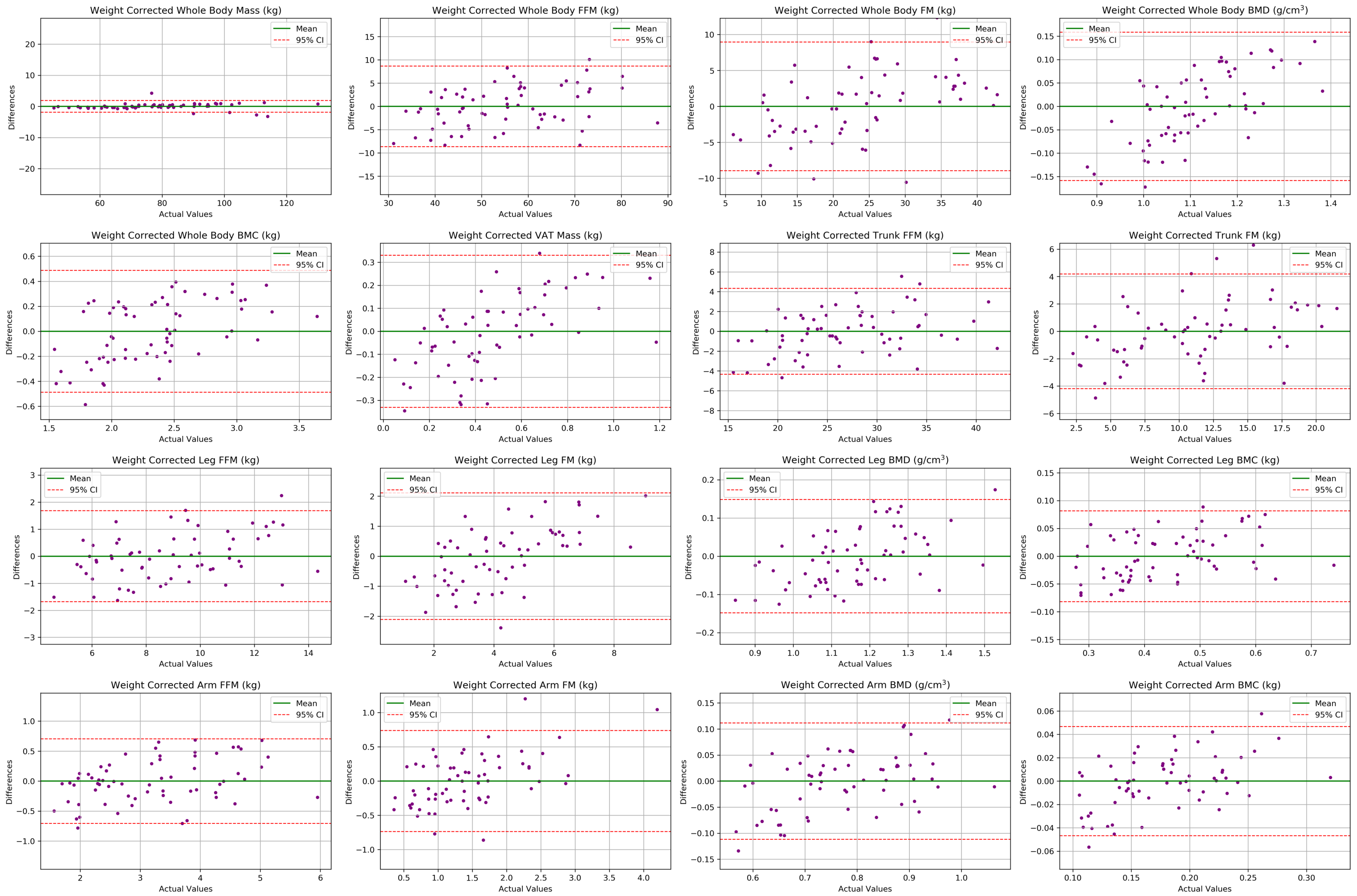

Supplemental Figure 5 Bland-Altman plots for actual DXA vs weight corrected Pseudo-DXA measurements of fat mas (FM), fat free mass (FFM), bone mineral content (BMC) and bone mineral density (BMD).

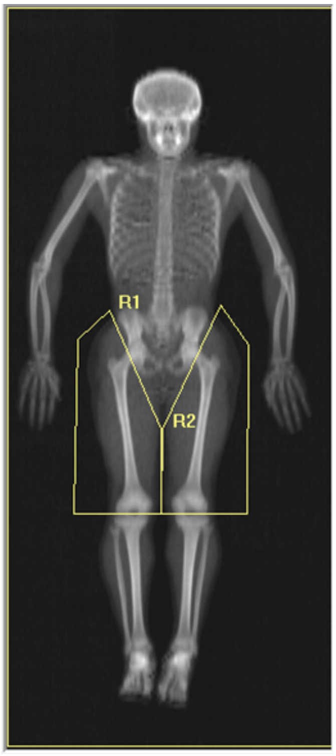

Supplemental Figure 6. Example DXA scan with regions of interest for defining the right thigh subregion (R1) and the left thigh subregion (R2)

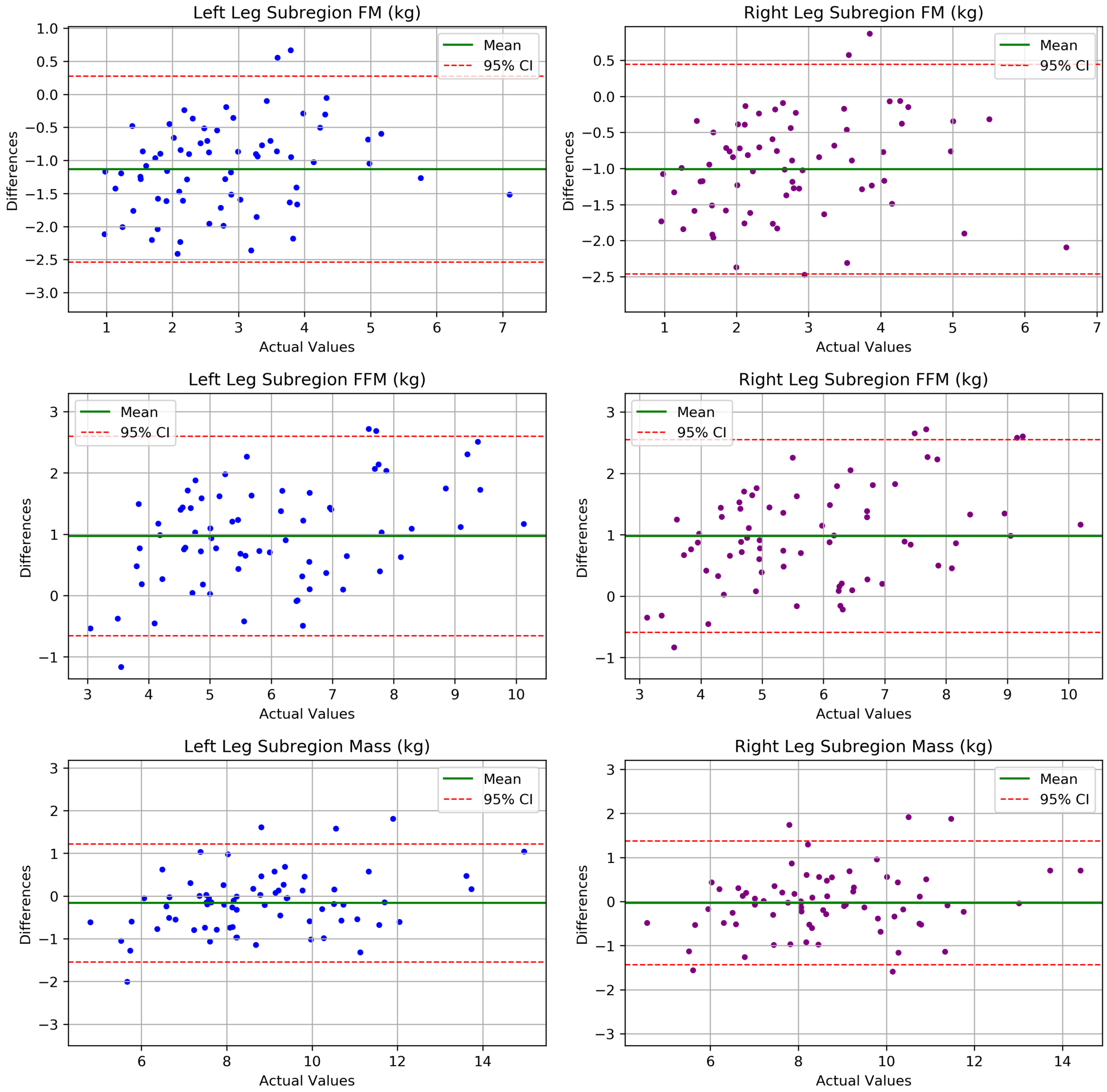

Supplemental Figure 7 Bland-Altman plots for actual DXA vs Pseudo-DXA measurements of fat mas (FM), fat free mass (FFM), and total mass for subregion 1
