# Supplementary material for: Generative deep learning furthers the understanding of local distributions of fat and muscle on body shape and health using 3D surface scans
Source: Commun Med (Lond). 2024 Jan 30;4:13. doi: 10.1038/s43856-024-00434-w (PMC10824755; doi:10.1038/s43856-024-00434-w)
Supplement: Supplementary file 2 — Reporting Summary [file 43856_2024_434_MOESM2_ESM.pdf]

nature portfolio

Corresponding author(s): John Shepherd

Last updated by author(s): Dec 14, 2023

Reporting Summary

Nature Portfolio wishes to improve the reproducibility of the work that we publish. This form provides structure for consistency and transparency in reporting. For further information on Nature Portfolio policies, see our Editorial Policies and the Editorial Policy Checklist.

Statistics

For all statistical analyses, confirm that the following items are present in the figure legend, table legend, main text, or Methods section.

n/a	Confirmed	
☐	☒	The exact sample size (*n*) for each experimental group/condition, given as a discrete number and unit of measurement
☐	☒	A statement on whether measurements were taken from distinct samples or whether the same sample was measured repeatedly
☐	☒	The statistical test(s) used AND whether they are one- or two-sided<br>*Only common tests should be described solely by name; describe more complex techniques in the Methods section.*
☐	☒	A description of all covariates tested
☐	☒	A description of any assumptions or corrections, such as tests of normality and adjustment for multiple comparisons
☐	☒	A full description of the statistical parameters including central tendency (e.g. means) or other basic estimates (e.g. regression coefficient) AND variation (e.g. standard deviation) or associated estimates of uncertainty (e.g. confidence intervals)
☐	☒	For null hypothesis testing, the test statistic (e.g. *F*, *t*, *r*) with confidence intervals, effect sizes, degrees of freedom and *P* value noted<br>*Give P values as exact values whenever suitable.*
☒	☐	For Bayesian analysis, information on the choice of priors and Markov chain Monte Carlo settings
☒	☐	For hierarchical and complex designs, identification of the appropriate level for tests and full reporting of outcomes
☒	☐	Estimates of effect sizes (e.g. Cohen's *d*, Pearson's *r*), indicating how they were calculated

Our web collection on statistics for biologists contains articles on many of the points above.

Software and code

Policy information about availability of computer code

Data collection	The Meshcapade GmbH, Tübingen, Germany, was used to standardize and pose the 3D body scans. Hologic, Inc Apex version 5.5 software was used to derive body composition measures from real and model generated DXA scans.
Data analysis	Custom code which details the final model architecture will be versioned and available for download via Github and Zenodo upon publication.

For manuscripts utilizing custom algorithms or software that are central to the research but not yet described in published literature, software must be made available to editors and reviewers. We strongly encourage code deposition in a community repository (e.g. GitHub). See the Nature Portfolio guidelines for submitting code & software for further information.

Data

Policy information about availability of data

All manuscripts must include a data availability statement. This statement should provide the following information, where applicable:

- Accession codes, unique identifiers, or web links for publicly available datasets
- A description of any restrictions on data availability
- For clinical datasets or third party data, please ensure that the statement adheres to our policy

The Health ABC is available from the National Institute on Aging, but restrictions apply to the availability of these data, which were used under license for the current study, and so are not freely available. Data however can be requested through the study's website at healthabc.nia.nih.gov. The BMDCS data can be accessed through the NICHD DASH website (https://dash.nichd.nih.gov/). The Shape Up! Adults is available from the corresponding author upon reasonable request

or at https://shapeup.shepherdresearchlab.org/for-researchers/. Final model weights and image processing software can be made available upon reasonable request to the corresponding author and through a data sharing agreement.

Human research participants

Policy information about studies involving human research participants and Sex and Gender in Research.

Reporting on sex and gender	We evaluated our final models performance on an unseen holdout test set. We report performance on the whole test set and we also report performance analysis based on sex. Sex was determined through self reporting and information on gender was not collected for this study.
Population characteristics	The self-supervised learning data set was sourced from two studies; Health, Aging, and Body Composition (Health ABC) and Bone Mineral Density in Childhood Study (BMDCS). The Health ABC study is a prospective cohort study of 3075 individuals (48.4% male, 51.6% female) aged 70 to 79 years at the time of recruitment, 41.6% of whom are Black with the remaining 58.4% being non-Hispanic White. The BMDCS is also a prospective study cohort of 2014 individuals (49.3% male, 50.76% female) aged 5-20 years.<br><br>The final model was trained on data sourced form the Shape Up! Adults study. This study is a cross-sectional study of healthy adults. Participants were recruited at Pennington Biomedical Research Center (PBRC), University of Hawaii Cancer Center (UHCC), and University of California, San Francisco (UCSF). Recruitment was designed to result in a diverse population that is well stratified by sex, age, ethnicity, and body mass index (BMI).
Recruitment	Recruitment for final model training was designed to result in a diverse population that is well stratified by sex, age, ethnicity, and body mass index (BMI).
Ethics oversight	The Heath ABC protocol was approved by the institutional review boards (IRB) at each field center (University of Pittsburg, PA and University of Tennessee, Memphis, TN), the BMDCS protocol was approved by the IRB at each clinical center (The Children's Hospital of Philadelphia, Cincinnati Children's Hospital Medical Center, Creighton University, Children's Hospital Los Angeles, and Columbia University) and the data coordinating center (Clinical Trials and Survey Corporation). The Shape Up! Adults protocol was approved by the Institutional Review Boards (IRBs) at PBRC, UCSF, and the University of Hawaii Office of Research Compliance.

Note that full information on the approval of the study protocol must also be provided in the manuscript.

Field-specific reporting

Please select the one below that is the best fit for your research. If you are not sure, read the appropriate sections before making your selection.

☒ Life sciences ☐ Behavioural & social sciences ☐ Ecological, evolutionary & environmental sciences

For a reference copy of the document with all sections, see nature.com/documents/nr-reporting-summary-flat.pdf

Life sciences study design

All studies must disclose on these points even when the disclosure is negative.

Sample size	Power and sample size calculations were performed prior to the submission of the grants from which the data for this manuscript reports on. The sample size of the self-supervised learning data was determined by the availability of full resolution whole body DXA data from the Health ABC and BMDCS studies. The sample size for the 3D to DXA modeling was determined by the ability to curate data pairs from each patient consisting of fully registered and pose standardized 3D body scans and full resolution whole body DXA body scans.
Data exclusions	DXA scan data was excluded if it was not the full resolution. DXA scans may be smaller than the full resolution if the scan was preset, prior to scanning, to be a different size of if the scan technician stopped the scan prematurely. While these scans may still be clinically valid, our model was designed to handle the full standard resolution and scans of smaller resolutions were excluded. For the 3D to DXA modeling specifically, patients were excluded if either their 3D body scan or DXA scan was not valid or missing. 3D scans were excluded if they were not or unable to be registered and standardized with the Meshcapade software
Replication	Machine learning models were validated on a hold out, unseen, test set. This mitigates overfitting and enables a level of reproducibility in that our final models are generalizable and our reported performance results are not inflated. Final models will also be made publicly available.
Randomization	The data was randomly split into a train, validation, and test set by patient ID for both the self-supervised learning phase and the 3D to DXA modeling phase. Splitting the data by patient ID was used to combat data leakage or the possibility that highly correlated data points from one patient does not end up in more than one sub data set. Random splitting of the data was also performed under the constraint that each sub dataset (train, validation, and test) contain equal proportions of cancer and benign patients. Therefore the distribution of outcomes in each subset is representative of the overall study population.
Blinding	Blinding was not applicable to this study

Reporting for specific materials, systems and methods

We require information from authors about some types of materials, experimental systems and methods used in many studies. Here, indicate whether each material, system or method listed is relevant to your study. If you are not sure if a list item applies to your research, read the appropriate section before selecting a response.

Materials & experimental systems

n/a	Involved in the study
☒	☐ Antibodies
☒	☐ Eukaryotic cell lines
☒	☐ Palaeontology and archaeology
☒	☐ Animals and other organisms
☐	☒ Clinical data
☒	☐ Dual use research of concern

Methods

n/a	Involved in the study
☒	☐ ChIP-seq
☒	☐ Flow cytometry
☒	☐ MRI-based neuroimaging

Clinical data

Policy information about clinical studies

All manuscripts should comply with the ICMJE guidelines for publication of clinical research and a completed CONSORT checklist must be included with all submissions.

Clinical trial registration	Not applicable/Not a clinical trial
Study protocol	Not applicable/Not a clinical trial
Data collection	Not applicable/Not a clinical trial
Outcomes	Not applicable/Not a clinical trial
